# Successful treatment with hyperbaric oxygen therapy for pneumatosis cystoides intestinalis as a complication of granulomatosis with polyangiitis: a case report

**DOI:** 10.1186/s13256-017-1421-1

**Published:** 2017-09-17

**Authors:** Kensuke Nakatani, Takaharu Kato, Shinichiro Okada, Risa Matsumoto, Kazuhiro Nishida, Hiroyasu Komuro, Toshiyuki Suganuma

**Affiliations:** 1 Department of Surgery, Yokosuka General Hospital Uwamachi, 2-36 Uwamachi, Yokosuka, Kanagawa 238-8567 Japan; 2 Department of Surgery, Saitama Medical Center, Jichi Medical University, 1-847 Amanuma-cho, Omiya-ku, Saitama 330-8503 Japan

**Keywords:** Granulomatosis with polyangiitis, Pneumatosis cystoides intestinalis, Intestinal perforation, Hyperbaric oxygen therapy, Case report

## Abstract

**Background:**

Although gastrointestinal involvement in patients with granulomatosis with polyangiitis is uncommon, it is associated with mild to severe life-threatening complications. We present a case of pneumatosis cystoides intestinalis in a patient with granulomatosis with polyangiitis that was treated successfully with hyperbaric oxygen.

**Case presentation:**

A 70-year-old Japanese man with a 3-year history of granulomatosis with polyangiitis consulted our hospital with a complaint of severe back pain. Computed tomography showed a large amount of gas located in his bowel wall and mesentery. He underwent urgent exploratory laparotomy, which led to a diagnosis of pneumatosis cystoides intestinalis without intestinal perforation or necrosis. He consequently underwent 13 sessions of hyperbaric oxygen therapy and was discharged from our hospital without complications.

**Conclusions:**

Several previous reports have supported the efficacy of hyperbaric oxygen for treating pneumatosis cystoides intestinalis. The present case, however, is the first in which pneumatosis cystoides intestinalis in a patient with granulomatosis with polyangiitis was successfully treated with hyperbaric oxygen. We therefore suggest that hyperbaric oxygen therapy could be a candidate treatment for pneumatosis cystoides intestinalis in patients with granulomatosis with polyangiitis.

## Background

Granulomatosis with polyangiitis, also known as Wegener granulomatosis, is a small-vessel vasculitis that predominantly affects the upper and lower respiratory tract and the kidneys. Diarrhea, bleeding, and/or perforation have been reported as gastrointestinal complications of granulomatosis with polyangiitis [1]. Although gastrointestinal involvement in patients with granulomatosis with polyangiitis is rather uncommon, it is sometimes associated with mild to severe life-threatening complications [2]. Pneumatosis cystoides intestinalis is defined as the presence of gas within the gastrointestinal tract wall. When acute complications appear (for example, perforation, peritonitis, necrotic bowel), surgery is indicated [3]. We present a case of pneumatosis cystoides intestinalis in a patient already diagnosed with, and treated for, granulomatosis with polyangiitis. He was treated successfully with hyperbaric oxygen (HBO) therapy for the pneumatosis cystoides intestinalis.

## Case presentation

A 70-year-old Japanese man had a 3-year history of granulomatosis with polyangiitis that had manifested as multiple nasal nodules, pulmonary cavities, and serum antineutrophil cytoplasmic antibody-positive, necrotizing, crescentic glomerulonephritis. He had taken 15 mg prednisolone and 150 mg of mizoribine per day since the diagnosis. Cyclophosphamide or rituximab was not used. Three years after the diagnosis (at which point the total amount of prednisolone was 17.5 g), he complained of severe back pain and consulted our hospital (Yokosuka General Hospital Uwamachi).

**Fig. 1:**
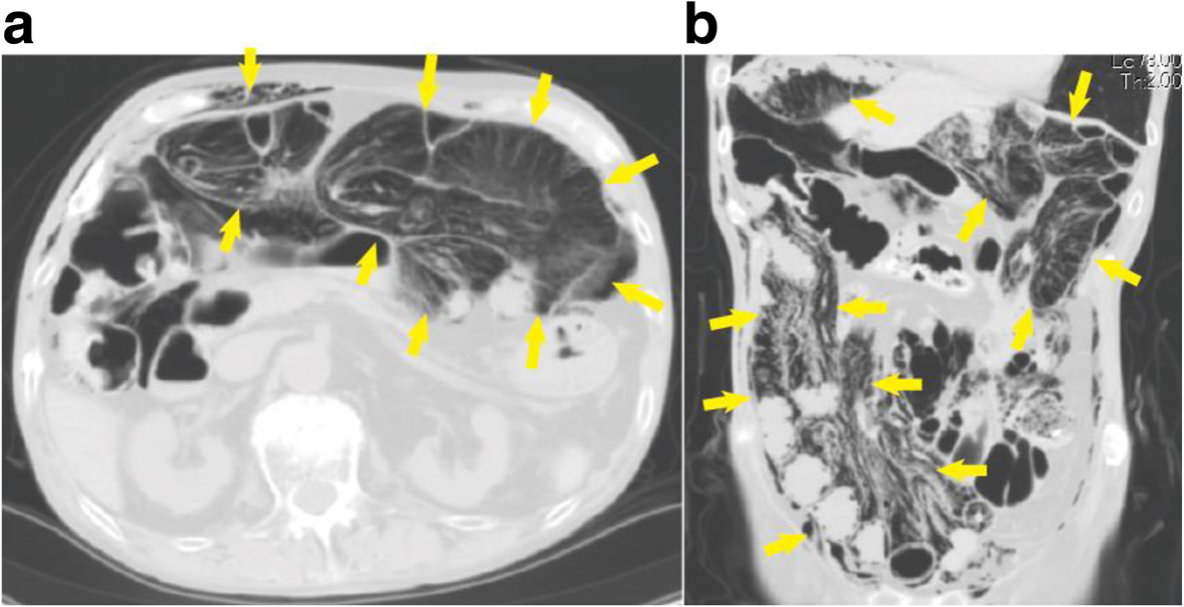
Computed tomography shows a large amount of gas in the abdominal wall, bowel wall, and mesentery (*arrows* in axial view (**a**) and in coronal view (**b**)). It was difficult, however, to rule out the existence of free air in the abdominal cavity. Portomesenteric pneumatosis was not detected

A physical examination revealed that his blood pressure was 143/93 mmHg, pulse rate 93 beats/minute, respiratory rate 16/minute, and body temperature 36.6 °C. His bowel sounds indicated hypoactivity, and his abdomen was distended but without tenderness. Laboratory analysis showed a C-reactive protein level of 12 mg/dl (normal 0 to 0.3 mg/dl) and white blood cell count of 9700/μl. Blood gas analysis showed pH 7.378, base excess − 6.0 mmol/L, and lactate 1.1 mmol/L. Computed tomography (CT) showed a large amount of gas located in his abdominal wall, bowel wall, and mesentery (Fig. 1). It was difficult, however, to rule out the possibility of intestinal perforation or necrosis.

**Fig. 2:**
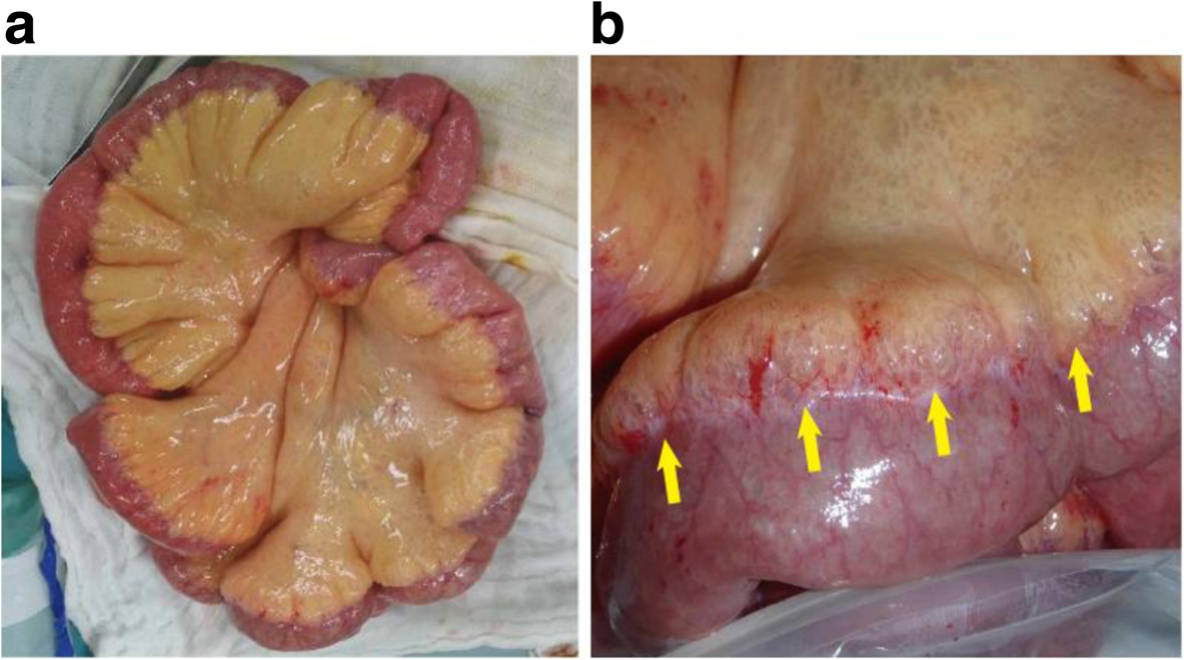
Intraoperative exploratory laparotomy findings (Overall view (**a**)) revealed pneumatosis intestinalis without intestinal perforation or necrosis (*arrows* in (**b**))

**Fig. 3:**
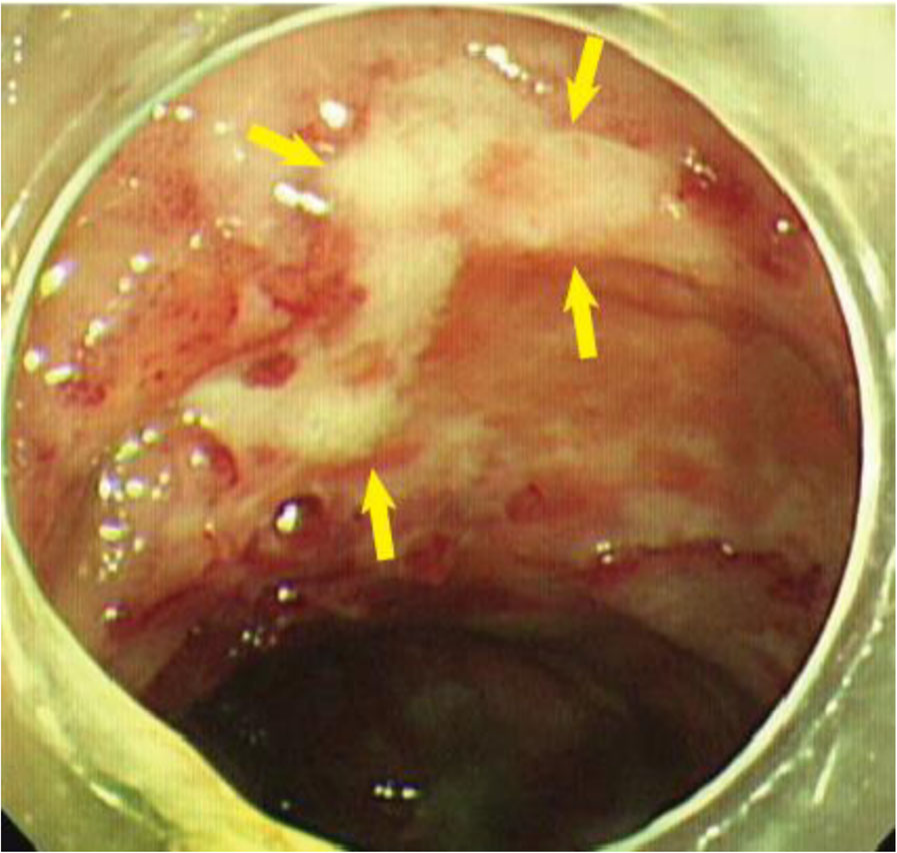
The colonoscopy which was performed 2 years before the diagnosis of pneumatosis cystoides intestinalis showed multiple ulcers in his colon on the right side (*arrows*)

**Fig. 4:**
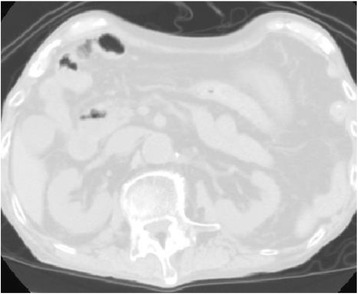
Computed tomography after hyperbaric oxygen treatment shows no gas located in the abdominal wall, bowel wall, or mesentery

He underwent an urgent exploratory laparotomy, which led to a diagnosis of pneumatosis cystoides intestinalis without intestinal perforation or necrosis (Fig. 2). Two years before this diagnosis, multiple ulcers had been detected in his colon on the right side (Fig. 3), so we surmised that some of these ulcers had developed pneumatosis cystoides intestinalis. The day after the exploratory laparotomy, he started HBO therapy at 2.0 atmospheres absolute (ATA) for 90 minutes/day. The treatment duration was 17 days, with 13 sessions. Following HBO therapy, we found no evidence of pneumatosis cystoides intestinalis on CT scans (Fig. 4). He was discharged from our hospital without complications 19 days after completion of the HBO treatment. At his most recent follow-up, 3 years after being diagnosed with pneumatosis cystoides intestinalis, he was clear of the disease.

## Discussion

We report the case of a patient with granulomatosis with polyangiitis who later developed pneumatosis cystoides intestinalis and underwent successful HBO treatment. To the best of our knowledge, this is the first case of pneumatosis cystoides intestinalis secondary to granulomatosis with polyangiitis that has been successfully treated with HBO.

Pneumatosis cystoides intestinalis is an uncommon disease with an unknown etiology. It is characterized by the presence of gas within the submucosa or subserosa of the intestine [3–5]. Gastrointestinal involvement is not common in patients with granulomatosis, although there have been some reports of multiple ulcerations and intestinal perforation in patients with granulomatosis with polyangiitis [6–10]. Gagliardi *et al.* found mucosal and histologic abnormalities in patients with granulomatosis with polyangiitis [11]. Pneumatosis cystoides intestinalis has also been interpreted as evidence of intestinal ischemia and impending perforation [12].

HBO has long been recognized as an effective therapy for pneumatosis cystoides intestinalis, leading to cyst regression (determined by imaging) and symptom resolution [13]. A number of case reports have been published supporting the efficacy of HBO for treating pneumatosis cystoides intestinalis [14]. Standard HBO treatment is 2 to 3 ATA for 6 to 90 minutes/day for a duration ranging from 3 to 33 days [14]. In the present case, HBO effectively cured pneumatosis cystoides intestinalis in a patient with granulomatosis with polyangiitis, without complications.

## Conclusion

We present a case of pneumatosis cystoides intestinalis in a patient with granulomatosis with polyangiitis that was treated successfully with HBO.

## Abbreviations

ATA: Atmospheres absolute
CT: Computed tomography
HBO: Hyperbaric oxygen
